# A multi-method study evaluating the inference of compartmental model parameters from a generative agent-based model

**DOI:** 10.1016/j.idm.2025.10.002

**Published:** 2025-10-16

**Authors:** Elizabeth Hunter, Jim Duggan

**Affiliations:** a Insight Centre for Data Analytics, University of Galway, University Road, Galway, H91 TK33, Ireland; b School of Computer Science, University Road, Galway, H91 TK33, Ireland

**Keywords:** Hamiltonian Monte Carlo, Nelder-Mead, SEIR, Calibration

## Abstract

Calibrating process models such as compartmental SIR Models to real data can be performed using either optimization or Bayesian techniques. To accurately assess the performance of these methods, synthetic outbreak data can be used. All information about the data generative process is known for synthetic data, while when using real data there are many unknowns such as under-reporting of cases or real parameter values. We propose using an agent-based model to generate synthetic data. Calibrating to synthetic datasets created using different agent contact structures can provide us with information on how changes in contact structures impact SIR model parameters. We compare results for two calibration methods: Nelder-Mead an optimization technique and HMC, a Bayesian technique. The analysis finds that the two calibration methods perform similar in terms of accuracy when looking at the Mean Absolute Error, Mean Absolute Scaled Error, and Relative Root Mean Squared Error. Looking at the model parameters, HMC is better able to capture the ground truth parameters then Nelder-Mead. The results of the calibration additionally show that the effective infectious period is sensitive to the changes in contact patterns and the proportion of susceptible individuals in the population. For choosing a calibration method, if overall accuracy is the desired outcome, either method should perform equally well, however, if the aim is to understand and analyse the model parameters HMC is a better choice. Understanding how the effective parameters such as the infectious period changes as contact patterns and vaccination rates change can provide valuable information in understanding how to interpret parameters calibrated from real world data that captures both isolation and vaccination.

## 1 Introduction

When calibrating a model to real data, there are two standard approaches: optimization and Bayesian calibration (Venkatramanan et al., 2018). In optimization, the difference between the observed and modeled data is minimized using a direct search (Tahmasebi et al., 2012). In Bayesian calibration, information about a system or a prior distribution is used in conjunction with the likelihood of the data to determine the probability of the data occurring given the model. The distribution of these probabilities is called the posterior distribution (Ellison, 2004). There are a number of challenges in calibrating models to real data, including incomplete data, multiple data sources, and changes in behaviours and policy that might impact the data (Swallow et al., 2022). We can understand how well our model fits to the reported data, but we do not know how well the model fits to the complete data (reported and unreported) because we do not know how many cases are unreported. Additionally, we do not know the underlying parameter distributions of the system.

To model the spread of an infectious disease an SIR compartmental model is often used. A compartmental model is one where the population is split into groups or compartments and an equation represents the movement of the population between each compartment (Brauer, 2008b). An SIR compartmental model has three compartments: Susceptible, Infected and Recovered (Kermack & McKendrick, 1927). Additional compartments can be added to include more detail in the model, such as Exposed, Asymptomatic or Vaccinated (Hethcote, 2000, pp. 599–653). The typical SEIR model has three main parameters that drive its dynamics: the *transmission rate*, the *latent* or *exposed period* and the *infectious period* (Kermack & McKendrick, 1927). These parameters can be drawn from the literature or the model can be calibrated to real data to find estimates. The models can also be deterministic, where every model run results in the same model output, or stochastic, where noise is introduced in the model and each model run results in different model output (Nåsell, 2002).

The parameters used in SIR type models are often defined for a homogeneous, fully susceptible population. For example, the basic reproduction number is the number of secondary cases from a primary case assuming a fully susceptible and homogeneously mixing population. These assumptions are not typically true of what happens in a real outbreak. Thus the effective reproduction number, *R*_*t*_, is often calculated (Nishiura & Chowell, 2009, pp. 103–121). *R*_*t*_ is the expected number of secondary cases from a primary case in a population that is not fully susceptible (Gostic et al., 2020). Because *R*_*t*_ takes into account the level of susceptibility of the population, the value of *R*_*t*_ will decrease as the number of susceptibles decreases, signifying a slower transmission when a small portion of the population is susceptible (Delamater et al., 2019; Ridenhour et al., 2014).

Other parameters might, however, be influenced by these fully susceptible and homogeneous mixing assumptions. For example, the infectious period has a biological definition of the actual number of days that an individual is infectious and an effective definition where it is the number of days a person is able to pass on the virus to another person (Vynnycky & White, 2010). If the infectious person isolates for part of their biological infectious period, they are reducing their effective infectious period (Becker et al., 2005). Other interventions such as mask wearing which reduces the amount of time an individual is shedding a virus can also lead to a reduction in the effective infectious period (Yang et al., 2021). As this happens often in real world scenarios, when calibrating an equation-based model to real data, the resulting parameters might be the effective parameters for the situation where the data was collected and not applicable if the contact patterns, interventions, and immunity rates of the population change. For example, if a model is calibrated to data from a situation where there is a lockdown or when cases are isolated earlier, the infectious period will likely be lower in the lockdown and earlier isolation scenarios compared to freer movement scenarios as the infectious individuals do not have as much effective time to infect others (Xin et al., 2023).

When using real world data, we do not have access to a large amount of information including the contact networks, behaviour changes, susceptibility of the population, and real disease parameters (Swallow et al., 2022). It is, therefore, difficult to untangle how changes in behaviours and population susceptibility might impact the predicted parameters. Synthetic data from models simulating outbreaks can be useful in creating a scenario where all conditions are known. Compartmental models have been used to generate such synthetic data (Andrade & Duggan, 2023). In these cases although noise is added to the incidence data produced from the model, e.g. Poisson or negative binomial noise, the data is generated from a system that has the same structure as the model that is being calibrated. We propose using an alternative method for synthetic data generation, an agent-based model. Agent-based models are computer simulations that comprise agents in an environment. The agents can interact with each other and their environment based on a set of coded rules and each agent can be given their own unique set of characteristics (Gilbert, 2008). They are useful tools for studying the impacts of individual behaviors on a larger social phenomenon. As they can include heterogeneous mixing, agent-based models have been found particularity useful in modelling infectious disease spread (Bruch & Atwell, 2015). Agent-based models have been used to simulate the spread of a number of different diseases including COVID-19 (Bilal et al., 2025; Hunter & Kelleher, 2022; Kerr et al., 2021), Mpox (Ajmal et al., 2024), tuberculosis (Bozzani et al., 2023) and cholera (Crooks & Hailegiorgis, 2014). Using an agent-based model instead of a compartmental model to generate synthetic outbreak data allows us to assess model calibration when data generated from a higher complexity system is estimated with a less complex model. Although an agent-based model remains a simplification of the real world, it allows for the generation of synthetic data that is driven by heterogeneous interactions and movements. Knowing the ground truth data from the agent-based model, an SEIR model can be calibrated to the agent-based ground truth data allowing for a greater understanding of calibration methods and their application to infectious disease models. The aim of our work is two-fold: The first to understand what methods of model calibration are most accurate compared to the ground truth case data when all information is known and the second to understand how changing contact patterns can impact the effective parameters of an infectious disease.

## 2 Methods

**Fig. 1:**
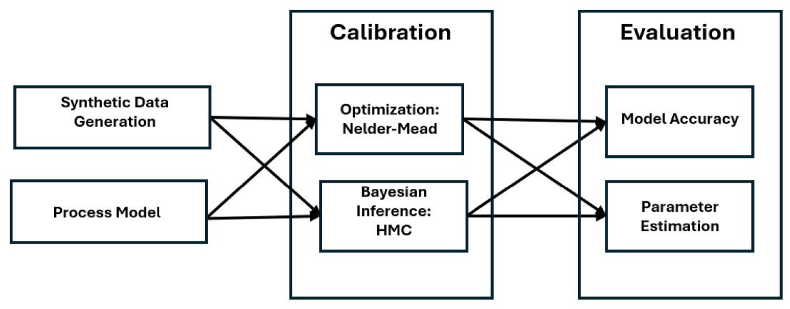
Outline of the experimental design of the paper. An agent-based model will be used to generate ground truth data and two calibrations will be explored.

To investigate the accuracy of different calibration methods and how changes in contact patterns impact effective disease parameters, an agent-based model is used to generate synthetic ground truth datasets of infectious disease outbreaks for different vaccination and social isolation scenarios. A deterministic process model is developed to reproduce the synthetic ground truth data. The process model considered is a compartmental SEIR model with two unknown parameters relating to the infectious period ($\frac{1}{\gamma }$) and transmission rate (*β*). Two estimation models or statistical inference methods, one optimization and one Bayesian calibration, are then used to calibrate the ground truth data sets to a deterministic process model. The optimization method used is Nelder-Mead (Nelder & Mead, 1965) with bootstrapping (Dogan, 2007), and the Bayesian method is Hamiltonian Monte Carlo (Duane et al., 1987). The mean absolute scaled error, the mean absolute error, and the relative root mean squared error are used to compare the calibrations across the different methods to determine which method produces the closest results to the ground truth. Then the distributions of parameters produced from the two inference methods are compared to determine how the parameters change based on different levels of social mixing and different levels of population susceptibility. Fig. 1 provides an overview of the experimental design and in the next sections more detail is provided on the agent-based model, the SEIR model, and the calibration methods.

### 2.1 Agent-based model

The model used to generate synthetic data for our calibration has been previously presented in (Hunter et al., 2018). It is an agent-based model for the spread of measles in Irish towns. Agent-based models for infectious disease spread have four main components that define the model (environment, society, transportation, and disease) (Hunter et al., 2017). Although the details of the model have been published previously, here we provide a brief description of each component.

#### 2.1.1 Environment

The model environment represents the town of Tramore in Ireland. Geographic data from the Irish census (CSO, 2014) is used to build the town. Each town is split into small areas, the smallest geographic area over which the Irish census is aggregated. Each small area contains between 50 and 200 dwellings. In addition to census data, data from open street maps (OpenStreetMap contributors, 2017) and myplan. ie (Myplan.ie, 2017) are used to determine the areas in the town that are residential, commercial, industrial and community. These areas will determine where agents in the model live, work and socialize. A set number of workplace locations are set in the commercial and industrial areas of the town. Data from the department of education provides the exact location of each primary and secondary school in the towns (2017).

#### 2.1.2 Society

The society of the model defines the agents that populate the model. Our society is built to match the Irish population in the town. We use census data (CSO, 2014) to determine the number of people in each small area of a town and populate the small area with the corresponding number of agents. Within a small area census data is used so that the correct proportion of agents are each age, sex, and economic status (working, student, retired, and unemployed). Households are also created to match the household structure in the small areas including the number of individuals in each household, the number of adults in households, number of children and ages of children (under 15, over 15, under and over 15). Households are assigned a household location in one of the residential areas of the town. Students are assigned to a primary or secondary school based on their age and location: if there are more than one primary or secondary school in the town agents will choose the school closest to their household location. Working agents choose one of the workplace locations in the town.

#### 2.1.3 Transportation

To move around the agents move in a straight line between their current location and desired destination taking steps along the way. If while moving between locations or when at a destination location agents share the same physical space as another agent, they will come into contact with them. Agents choose a destination in two ways: scheduled movements and random movements. Scheduled movements occur for agents who are students and working. Students and working agents will move between home and work/school and work/school and home at predefined times in the day. Random movements occur on the weekend for students and working agents, summer holidays for students and every day for agents who are retired or unemployed. For random movements, during daytime hours agents will pick a desired location from the possible community and residential areas in the town and move there. During nighttime hours in the model, if an agent is not at their home location they will move to the home location. If an agent is infectious they decide to stay home and self isolate or not based on a specified parameter.

#### 2.1.4 Disease

The model simulates the spread of measles through a town. At the outset a set number of agents start out as infected. If while moving throughout the town an infectious agent comes into contact with a susceptible agent, a probability will determine if the susceptible agent will be exposed.^1^ They will then be exposed but not infectious for a set period of time and then they will become infectious. The agent will be infectious for a given period of time where they can infect other agents and then will be recovered or immune and can not be reinfected. Vaccinations and prior immunity can be activated in the model and will reduce the likelihood that an agent will be infected if they come into contact with an infectious agent.

There are a number of disease related parameters that are needed to run the model. These parameters are taken from the literature. The exposed period of measles is approximately 10 days and the infectious period is approximately 8 days (Nelson & Williams, 2007). In the model when infected an agent will chose their exposed period from a normal distribution with an average of 10 and standard deviation of 0.5 and their infectious period from a normal distribution with an average of 8 and a standard deviation of 0.5. To determine the probability of an infectious agent infecting a susceptible agent on contact we use the formula:

(1) $$p=\frac{R_{0}}{cd}$$

Where *R*_0_ is the basic reproduction number (the number of secondary cases generated from a primary case in a disease free and completely susceptible population), *c* is average contacts per time step, *p* is infections per contact and *d* is time per infection (in time steps) (Thomas & Weber, 2001). For our model the only value that is unknown is *p*. For measles, *R*_0_, is thought to be between 12 and 18 (Nelson & Williams, 2007). For the agent-based model we set *R*_0_ to be 12, *d* is the time per infection which is on average 8 days for measles or 96 time steps in our model and *c* the average contacts per times step can be calculated directly from the model. All unvaccinated agents will have the same probability of infection. If an agent is vaccinated their probability of infection will be reduced by the vaccine effectiveness.

#### 2.1.5 Schedule

Agent-based models are run on discrete time steps. In the Irish town model each time step represents 2 hours in a day. On weekdays, agents who are students or workers will move between home and school or home and work at specified times. On weekends, school holidays for students, and any day for retired or unemployed agents, all agents move randomly through the residential and community areas of the town. The model runs until there are no more agents exposed or infected.

#### 2.1.6 Synthetic data generation

**Fig. 2:**
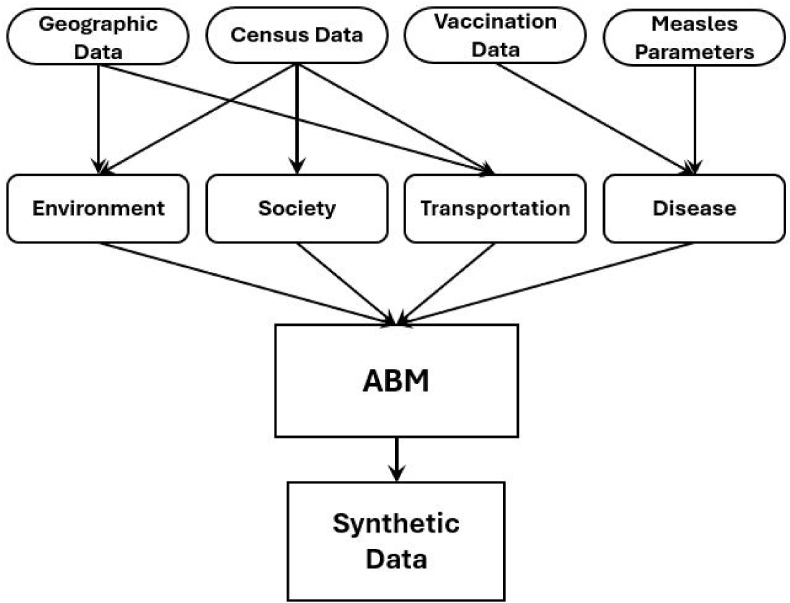
Outline of the process for creating our synthetic data.

Although a framework of using SEIR models as data generators has been widely applied (Andrade & Duggan, 2023), here we use an agent-based model to generate our synthetic data. This provides the facility to make changes to the agent behaviours, for example increased isolation or increased mixing, by adjusting parameters in the agent-based model while holding all other parameters constant. When an SEIR model is then calibrated to the synthetic data, any changes in parameters can be attributed to the change in the adjusted behaviours. Fig. 2 provides a visual overview of the synthetic data generating process further described below.

Agent-based models are stochastic in nature and each model run produces a different outbreak. Thus, it is necessary to run a model multiple times in order to capture a fuller distribution of the possible model output (Hunter & Kelleher, 2020). To generate the synthetic data for SEIR model calibration, we run the agent-based model 50 times with the same initial conditions. The model is set up to simulate a measles outbreak in the town of Tramore. There are 9547 agents in the model. When agents have immunity they have a 97 % chance of not becoming infected when exposed to an infectious agent. This corresponds with the effectiveness of the MMR vaccine for measles after two doses (CDC, 2024). The model starts with one agent infectious and the rest either susceptible and not vaccinated or susceptible and vaccinated. For each model run at each time step the number of agents who are susceptible, exposed, infectious, immune (from infection during the current outbreak), and newly infected that day are recorded. The model runs until there are no more cases in the population; thus the length of the outbreak varies by simulation run and some scenarios have longer temporal intervals. In the model there is no delay in reporting newly infected cases per day and all cases are reported (i.e. mandatory reporting of cases is assumed).

We generate nine datasets from our agent-based model to investigate the calibration methods, each dataset contains the output from 50 different model runs. The calibration datasets are generated using the same initial conditions but the values for the chance the agent stays home when sick and self isolates vary between each run. Three potential values for self isolation are considered: 0 %, 50 %, and 100 %. If the self isolation chance is 0 % agents will not stay home at all when infectious, if the value is 50 % agents have a 50 % chance of staying home each time step when they are infectious and if the self isolation chance is 100 % agents will stay home at all times when infectious. Three potential vaccination scenarios are considered: 0 % of the population is vaccinated, 50 % of the population is vaccinated and 80 % of the population is vaccinated. Vaccination rates of 0 % and 50 % for measles are unrealistic, especially within Ireland where the majority of the population over a certain age group would have been infected as children and the first quarter of 2024 vaccination rate for MMR1 in Ireland is 89.3 % (HPSC, 2025). However, specifying different vaccination rates allows for the understanding of how the fitted model parameters change as vaccination rates in a population increase. For the purpose of simplicity we do not consider age when choosing agents who are vaccinated: each agent is equally likely to be vaccinated regardless of if they are an adult or child. The combinations of the three self isolation and the three vaccination categories result in nine synthetic datasets described in Table 1.

**Table 1:** Scenarios.

Scenario	Scenario Name	Self Isolation Rate	Vaccination Rate
1	S1_I0_V0	0 %	0 %
2	S2_I50_V0	50 %	0 %
3	S3_I100_V0	100 %	0 %
4	S4_I0_V50	0 %	50 %
5	S5_I60_V50	50 %	50 %
6	S6_I100_V50	100 %	50 %
7	S7_I0_V80	0 %	80 %
8	S8_I50_V80	50 %	80 %
9	S9_I100_V80	100 %	80 %

When analysing the synthetic data sets, we found that there were runs, particularly in the 50 % vaccination scenarios where there were two peaks of measles cases. These two peaks are likely due to the contact network structure of the model where the outbreak is beginning to die out in one contact network but then moves to another pool of susceptible individuals. For simplicity we restrict the data to the first peak and do not calibrate to the second peak. This allows for us find the best calibration to the first peak without the noise of the second peak impacting calibration.

**Fig. 3:**
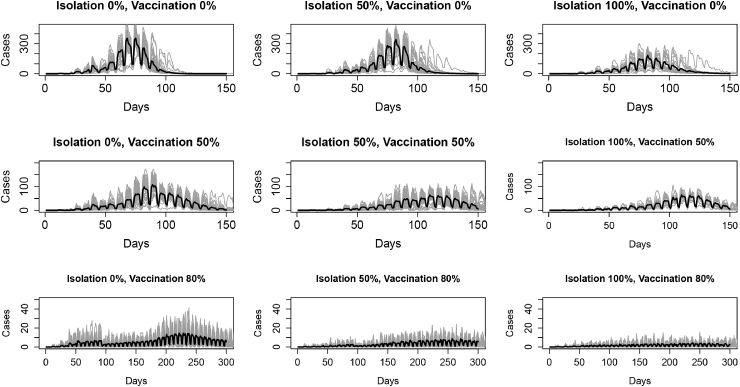
The incidence by day across 50 runs for each of the nine synthetic data sets generated from the Agent-Based Model. Individual runs are lines in grey. Average incidence by day across the 50 runs is in black. Note that the y scales of the plots are different for each row of the figure.

Fig. 3 shows the incidence for the each run for each of the nine synthetic datasets by day as well as the average incidence per day across the runs (only including the first peak of the outbreak). The average is taken by finding the average of each of the 50 model runs at each time step. The incidence curves are not smooth in some cases there is a large variation in the incidence between days. This is due to the stochastic nature of the agent-based model, where probabilities determine agent actions including infections which will lead to variations in cases, as well as the contact network structure of agents and agent schedules where agents interact with their school and work networks more during weekends resulting in fewer infections on certain days of the week.

### 2.2 Deterministic process model

As the underlying disease component of the agent-based model is related to an SEIR model, with agents moving from the states of Susceptible to Exposed, Infected and then Recovered, the model we calibrate using the synthetic data should be of a similar structure.

**Fig. 4:**
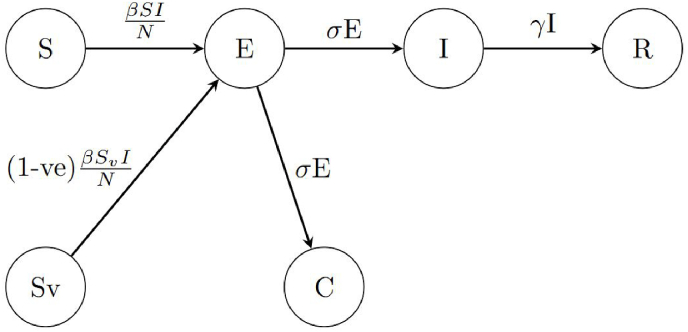
Flow between compartments in the deterministic process model.

Fig. 4 describes the flow between compartments for the process model used for our statistical inference. The model is a deterministic SVEIR compartmental model with compartments for Susceptible and Unvaccinated, Susceptible and Vaccinated, Exposed, Infected and Recovered. The equations for the model are:

(2) $$\frac{dS}{dt}=\frac{-\beta SI}{N}$$

(3) $$\frac{dS_{v}}{dt}=-\left(1-ve\right)\frac{\beta S_{v}I}{N}$$

(4) $$\frac{dE}{dt}=\frac{\beta SI}{N}+\left(1-ve\right)\frac{\beta S_{v}I}{N}-\sigma E$$

(5) $$\frac{dI}{dt}=\sigma E-\gamma I$$

(6) $$\frac{dR}{dt}=\gamma I$$

(7) $$\frac{dC}{dt}=\sigma E$$

Where S represents the unvaccinated susceptible, *S*_*v*_ are the vaccinated susceptible, *E* are the exposed but not infectious, *I* the infectious, *R* recovered and immune and *C* the total cases. N is the population size, *β* is the transmission rate, *ve* is the vaccination effectiveness, *σ* is one over the exposed or latent period and *γ* is the inverse of the infectious period. In the model, vaccinated individuals that become infected have the same *β* (transmission rate), *σ* (inverse of the exposed period) and *γ* (inverse of the infectious period) as the unvaccinated individuals. The transmission rate (*β*) for vaccinated individuals is, however, modified by the vaccine effectiveness such that the transmission rate is reduced by the multiplier (1 − *ve*). There is no movement considered between the recovered and susceptible compartments as measles is known to produce lifelong immunity after infection (Markowitz et al., 1990). Although our scenarios include isolation, we do not include a compartment for isolation in our deterministic process model as one of our research aims is to determine how the effective parameters of the model change with respect to changes in contact patterns. As we are generating the synthetic data with an agent-based model we know why contact patterns are changing in the model (e.g. isolation when infected) but in a real scenario the reason why contacts are increasing or decreasing might not be known. Therefore, it is important to use a deterministic process model without an isolation compartment as a way to understand how the parameters adapt to changes in contact patterns without added compartments.

### 2.3 Calibration methods

Two calibration methods are considered to obtain an understanding of how different methods might impact the results of the statistical inference of a model. The methods are Nelder-Mead an optimization technique and Hamiltonian Monte Carlo (HMC), a Bayesian technique. When calibrating we aim to find the optimal values for the transmission rate *β* and the inverse of the infectious period, *γ*. The initial conditions for our *β* and *γ* are those that are used in the agent-based model with *β* set to 1.5 and *γ* set to $\frac{1}{8}$. As it is possible to get solutions from an optimization method that are mathematically possible but biologically implausible we restrict the ranges searched for both *β* and *γ* so that our results are plausible. These restrictions are based on the ground truth parameters in the agent-based model as well a knowledge from the literature. For *β* we restrict values between 0.125 and 2.5 and for *γ* we restrict values between 0.1 and 1. This restricts the infectious period to between 1 and 10 days. Ten days was chosen as a maximum infectious period to correspond with the agent-based model where an agent is infectious for on average 8 days but their infectious period is drawn from a normal distribution with mean of 8 days and standard deviation of 0.5 days. While interventions implemented in the model will potentially reduce the effective infectious period they will not increase the effective infectious period. The restrictions on *β* and *γ* would also result in an *R*_0_ between 0.125 and 25 (using the relationship *R*_0_ = $\frac{\beta }{\gamma }$ (Keeling & Grenfell, 2000)). For measles *R*_0_ is known to be between 12 and 18 (Nelson & Williams, 2007). Thus our restricted range would allow for the contact networks and agent behaviours to both increase and decrease infectiousness. For the variables in the process model, which we are not finding through calibration (*σ*, and *ve*) we use the same values that are used in the agent-based model, with *σ* equal to $\frac{1}{10}$ and *ve* equal to 97 %. The vaccine rate is changed in the model based on the agent-based model scenario that is being used as the ground truth data. The methods are discussed in more detail below.

#### 2.3.1 Nelder-Mead method

The Nelder-Mead method is a minimization algorithm that uses a simplex approach to find the minimization of a cost function of n variables. A simplex of n + 1 vertices is created and the vertices in the simplex are adjusted using reflection, contraction, and expansion. New vertices are accepted if the resulting simplex produces a new minimum value for the function. The stopping criteria for adjusting the simplex is typically done by comparing the standard error of the cost function with a pre-selected value. Once the standard error falls below the pre-selected value the process stops (Nelder & Mead, 1965). There is also typically a maximum number of iterations or adjustments of the vertices that once reached will cause the method to stop. The Nelder-Mead method is independent of the gradient of the cost function and can be used for functions that are non-differentiable or when the gradient is not known. However, the method can be time consuming with more points needing to be evaluated and can run into problems near local minima where it might have difficulty converging (Maia et al., 2017). The method also relies heavily on initial conditions (Andrade & Duggan, 2020).

Within infectious disease epidemiology the Nelder-Mead method is widely used. In a comparison of Nelder-Mead, nonlinear regression and curve fitting, Conde-Gutierrez et al. (2021) find the Nelder-Mead method converged in the shortest possible time for fitting a Gompertz model for COVID-19 deaths in Mexico. The Nelder-Mead method has been used to fit an individual-based model in Nsoesie et al. (2013), where the Nelder-Mead algorithm is used to propose new parameter values which are then used in the individual-based model. They found that in most cases using this method the forecasted mean value converged to the true mean value, however, the initial parameters were crucial to the performance of the algorithm. Andrade and Duggan (2020) find that the Nelder-Mead is a reliable benchmark for estimating transmission rates when a broad choice of starting points are chosen. Despite the known shortcomings of the method and reliance on initial conditions, the Nelder-Mead method is used to optimize parameters for models for a number of diseases including COVID-19 (Ali et al., 2021), Marburg virus (Amoah-Mensah et al., 2024), HPV and cervical cancer (Taylor et al., 2010) and HIV (Zang et al., 2019).

To implement the Nelder-Mead method we use the *FME* package in R, a package designed to help fit models to data (Soetaert and Petzoldt, 2010). In particular we use the *modFit* function which allows for constrained optimization using the Nelder-Mead method. The process that is followed for the Nelder-Mead calibration is described below and summarized in Algorithm 1: Nelder-Mead and Bootstrap Process. The cost function we use for the Nelder-Mead method is the least squares function that minimizes the sum of the squared differences between the actual values and the predicted values. The equation for the cost function is:

(8) $$SSE=\overset{n}{\underset{i=1}{\sum }}{\left(Y_{i}-F_{i}\right)}^{2}$$

where n is the number of data points, *Y*_*i*_ is the ground truth data points and *F*_*i*_ is the predicted or forecasted data points from calibration. The Nelder-Mead algorithm on its own does not provide a distribution of possible parameter values as we would get from the HMC calibration. Therefore, in order to get a distribution of the optimized parameters, for each of the 50 ground truth data sets for each scenario we create 100 additional outbreak datasets using bootstraping. This method has previously been used by Mumtaz (2024) to compare HMC and Powell's method, another optimization method. This allows us to run the Nelder-Mead method 100 times for each ground truth. For each scenario this results in a distribution of 5000 optimized parameters. To perform the bootstrapping we follow residual-based nonparametric bootstrap used in Dogan (2007) and Mumtaz (2024). The method has five main in steps:

1. Optimize: The sum of squared difference between the synthetic data and the process model output is optimized.
2. Compute Error Terms: Error terms are calculated by subtracting the optimized model output from the synthetic data.
3. Nonparametric Resampling: Error terms are then resampled to create new error values for each time point. This is done by randomly drawing with replacement from the computed error terms in the previous step.
4. Create new data sets: A new data set is created by adding the resampled error terms to the model output using the initial conditions and original parameters values.
5. Optimize with new data set: We then use the new data sets and optimize the sum of squared difference between the new data set and the process model.

For each set of the 100 bootstrap samples from an individual ground truth, we calculate the 2.5 %, 5 %, 25 %, 50 %, 75 %, 95 % and 97.5 % quantiles for the parameter estimates to capture the distribution of the estimates. As each ground truth is from a separate iteration of the agent-based model, resulting in different outbreak scenarios, we treat each set of bootstrapped parameters as their own model and use an ensemble to combine the quanitles. Sherratt et al. (2023) found that a median ensemble is more robust to outliers than a mean ensemble. Therefore, for each quantile we find the median across the 50 ground truths and the resulting medians make up our overall Nelder-Mead parameter distributions. We also calculate a weighted median ensemble that gives more weight to the distributions that better fit the ground truth data. The weights used are the inverse of the normalized Mean Absolute Scaled Error (MASE).

The MASE is a measure of accuracy of forecasts (Franses, 2016). It is calculated by dividing the forecast error by the mean absolute error of the in-sample one-step naive forecast. Taking the absolute value of the measure and the mean across time points. The formula is:

(9) $$MASE=\frac{\frac{1}{n}\Sigma_{i=1}^{n}|Y_{t}-F_{t}|}{\frac{1}{n-1}\Sigma_{i=2}^{n}|Y_{i}-Y_{i-1}|}$$

where *Y*_*t*_ is the ground truth data at time t and *F*_*t*_ is the forecast of *Y*_*t*_. If the MASE is less than one it suggests that the forecast is better than the average one-step naive forecast computed in sample. If the MASE is grater than one it suggests the alternative, that the forecast is worse (Hyndman & Koehler, 2006).

For each of the 50 ground truth data sets, we find the MASE for each of the 100 sample parameters comparing the ground truth data to the output from the given sample parameters. The mean of the 100 MASE values is taken so that each distribution of sample parameters for one of the 50 ground truth data sets is associated with an MASE that estimates the average accuracy for all Nelder-Mead samples for the ground truth data set. The mean of the 50 MASEs is also taken to get an idea of how well on average all the samples fit to the data for each scenario.

Algorithm 1 Nelder-Mead and Bootstrap Process

**Table:** 

1: **for***Ground Truth Data Sets* = 1, 2, …50 **do** 2: Fit model to *Ground Truth Data Set* = *i* 3: **for***j* = 1, 2, …, 100 **do** 4: Create bootstrap sample using the fitted model and model error terms 5: Fit new model to bootstrap sample *j* using Nelder-Mead 6: Find *MASE* using *Ground Truth Data Set* = *i* and model fitted to bootstrap sample j 7: Calculate Control Reproduction Number *R*_*C*_ for model fitted to bootstrap sample j 8: **endfor** 9: Find mean *MASE* across the models fitted to the 100 bootstrap samples 10: Find quantiles for *β*, $\frac{1}{\gamma }$ and *R*_*c*_ 11: **endfor** 12: Find weighted median quantiles across 50 *Ground Truth Data Sets* for *β*, $\frac{1}{\gamma }$ and *R*_*c*_ weighting with the mean *MASE*

#### 2.3.2 Hamiltonian Monte Carlo

Hamiltonian Monte Carlo or HMC is a Bayesian inference method that can be used in parameter estimation. Bayesian inference is a method that uses information about a system that can be summarized in a quantitative model or prior distribution and the likelihood of data to determine the probability of a hypothesis or event (posterior probability). The posterior probability is presented in the form of a distribution and can provide a measure of the likelihood of the model given the data (Ellison, 2004). Simulation methods, in particular Markov Chain Monte Carlo (MCMC) algorithms, are widely used to provide estimates of the posterior distribution by drawing draw samples from the posterior distribution (Grinsztajn et al., 2021). MCMC is a method that generates sequential samples, where the previous sample values are used to generate the subsequent values, thus creating a Markov chain. A Markov chain is a sequence of random variables that are generated by a process where the current values of the process only depend on the previous values (Carlo, 2004). HMC is an MCMC algorithm that is used as an alternative to the early implementations of MCMC with Metropolis algorithms and Gibbs sampling as it suppresses the random walk behaviour in the earlier algorithms resulting in a more efficient search of the parameter space. HMC uses concepts from differential geometry and Hamiltonian dynamics in physics (Andrade & Duggan, 2020). Unlike the Nelder-Mead method which uses a traditional cost function, the parameter space in HMC is searched by simulating the movement of a frictionless particle over a surface determined by the likelihood function and the prior distribution (Andrade & Duggan, 2021). The movement of the frictionless particle is determined by the Hamiltonian function, the sum of the potential and kinetic energy of the system (Duane et al., 1987). This Hamiltonian function is the joint density and the equation for the joint density is:

(10) H(ρ,θ)=T(ρ|θ)+V(θ)

where, *θ* is the set of parameters and *ρ* is an auxiliary momentum variable. *T*(*ρ*|*θ*) is the kinetic energy and *V*(*θ*) is the potential energy. HMC is typically run for a set number of iterations. For each iteration, a set of steps are done. The momentum vector is sampled and the parameters are updated. A Metropolis acceptance step is then applied to determine if the new set of parameters should be kept or if the existing state should remain. The Metropolis acceptance step is:

(11) $$min\left(1,exp\left(H\left(\rho ,\theta \right)-H\left(\rho^{*},\theta^{*}\right)\right)\right)$$

where *ρ*∗ and *θ*∗ are the new parameters (Stan Development Team, 2024). There are a number of tuning parameters that are used in the HMC algorithm and the results are sensitive to these tuning parameters. The development of the statistical programming language and platform Stan and the No-Turn Sampler algorithm used by Stan has, however, resolved the issue of parameter tuning with HMC (Grinsztajn et al., 2021). Stan was created to provide a flexible probabilistic programming language for statistical modelling that includes a set inference tools for fitting models. It provides a full Bayesian inference for continuous variable models through MCMC including HMC and has packages and interfaces for command line, Python, R Matlab, Julia, Stata and Mathematica (Carpenter et al., 2017). With Stan a user can focus on modelling, while the statistical inference occurs under the hood and allows for the constraining of parameter priors based on background knowledge (Grinsztajn et al., 2021).

Similar to the Nelder-Mead method, HMC is used widely within the field of infectious disease epidemiology. Using HMC to calibrate age-structured cohort models (Andrade & Duggan, 2020) determined that HMC was able to provide similar estimates of parameters to other calibration methods while also being able to converge from dissimilar starting points, requiring less stringent assumptions for parameter uncertainty measurement and additionally HMC revealed correlations between parameters that could provide useful insight to decision makers. Stojanović et al. (2019) compare model calibration for a probabilistic model using HMC with a model calibration using a maximum likelihood approach for three different infectious diseases and show that the two approaches have similar performance. However, they find that the Bayesian HMC approach provides more information on model features and parameter distributions that would be considered valuable in policy making decisions.

HMC has been used in statistical inference for a number of infectious diseases and model types: including estimating transmission flows and sources of HIV infection in Africa with Stan (Xi et al., 2022), inferring the transmission probabilities for the 17th century plague (Ho et al., 2018), estimating infection rates for Ebola (Ho et al., 2018), investigating the impact of vaccination on model parameters during COVID-19 (Jdid et al., 2024) and updating constrained parameters in the spread of tuberculosis in wild badger populations (Konzen et al., 2024). Other methods of Bayesian inference such as MCMC are also commonly used in statistical inference for both compartmental SEIR models and agent-based models (Semochkina & Walsh, 2025).

We implement the HMC algorithm in R using Stan. The algorithm describing our process for running the HMC calibration and aggregating the output can be found below in Algorithm 2: HMC. For the two parameters that we are calibrating to, lognormal priors are used with *μ* equal to 0 and *σ* = 1. However, we restrict the values of *β* and *γ* that can be sampled to the same ranges as used in the Nelder-Mead calibration: *β* between 0.125 and 2.5 and *γ* between 0.1 and 1. We run 4 simultaneous chains of 1000 warm up iterations and 1000 iterations each. Running HMC for one of the agent-based model ground truths results in 4000 samples, meaning that for the 50 ground truths there are 200,000 samples. For each set of 4000 samples from an individual ground truth, we calculate the 2.5 %, 5 %, 25 %, 50 %, 75 %, 95 % and 97.5 % quantiles for the parameter estimates to capture the distribution of the estimates with the same methodology we use to aggregate the Nelder-Mead output described in Section 2.3.1, with a median weighted ensemble.

Algorithm 2 HMC

**Table:** 

1: **for***Ground Truth Data Sets* = 1, 2, …50 **do** 2: Setup *Stan* model: 3: Define data as *GroundTruthDataSet* 4: Define parameters and restrictions on parameters 5: Building and solve the process model to transform the parameters to produce the predictions for cases per day. 6: Define the model by computing the log joint density 7: Use *Stan* to run 4 chains of 1000 warm-up iterations and 1000 iterations 8: **for** Each iteration: **do** 9: **for** Each step: **do** 10: Run operations on parameters defined by the transformed parameters 11: Compute the log joint density 12: Use Metropolis acceptance to determine if proposed state or existing state is kept 13: **endfor** 14: Compute quantities of interest 15: **endfor** 16: Find *MASE* for each of the 4000 iterations comparing *Ground Truth Data Set*_*i*_ with output from the fit from each of the 4000 iterations. 17: Find mean *MASE* across the 4000 iterations 18: Calculate Control Reproduction Number *R*_*C*_ for each of the 4000 iterations 19: Find quantiles for *β*, $\frac{1}{\gamma }$ and *R*_*c*_ from the posterior distributions 20: **endfor** 21: Find weighted median quantiles across 50 *Ground Truth Data Sets* for *β*, $\frac{1}{\gamma }$ and *R*_*c*_ weighting with the mean *MASE*

#### 2.3.3 Evaluation

For each calibration method and each scenario we compare both inferred parameters and measures of model performance. The parameters that we find through calibration are *β*, the transmission parameter and *γ*, the inverse of the infection period. For ease of interpretation we convert the *γ* parameter to the infectious period. Additionally, as the transmission rate in an SEIR compartmental model is influenced by not only mixing rates but by the susceptible population, to more easily compare the transmission rates we calculate the control reproduction number *R*_*c*_. *R*_*c*_ is an alternative to the basic reproduction number *R*_0_ that can be used when the population is not fully susceptible. We adjust the formula for *R*_*c*_ from (Brauer, 2008a, pp. 321–348) to fit our model:

(12) $$R_{c}=\left(1-vr\right)*R_{u}+vr*R_{v}$$

where:

(13) $$R_{u}=\frac{S_{0}\beta }{N\gamma }=R_{0}$$

(14) $$R_{v}=\frac{ve*S_{0}\beta }{N\gamma }$$

In the above calculations *R*_*u*_ is the reproduction number for the unvaccinated or completely susceptible population and is equivalent to the basic reproduction number *R*_0_. *R*_*v*_ is the reproduction number for the vaccinated population and *vr* is the vaccination rate of the population.

To compare the performance of the models, we look at Mean Absolute Scaled Error (MASE), the Mean Absolute Error (MAE) and the Relative Root Mean Squared Error (RRMSE).

The formula for the MAE is:

(15) $$MAE=\frac{\Sigma_{i=1}^{n}|Y-F|}{n}$$

Where n is the number of observations in the time series, Y are the ground truth or actual values and *F* are the predicted or forecasted values. For each of the 50 ground truth data sets, we find the MAE for either each of the 100 sample parameters in the Nelder-Mead calibration or 4000 sample parameters in the HMC calibration comparing the ground truth data to the output from the given sample parameters. The mean of the MAE values is taken for all sample parameters. This results in each of the 50 ground truth data sets having their own MAE. The mean of the 50 MAE values, one for each ground truth, is also taken to understand how well on average all the samples fit to the data for each scenario. The MAE is useful measure for evaluating the performance of a model in predicting the forecast median and is robust to outliers. However, one of the main drawbacks of the MAE is that it is not scale free and is relative to the scale of the time series (Diamantis, Evangelos Spiliotis, & Assimakopoulos, 2022). As the agent-based model ground truth data is noisy, the robustness to outliers was also an important aspect in considering accuracy metrics. Additionally, for comparing calibrations for a single scenario, the scales of the outbreak sizes are the same. Thus, the MAE is useful in understanding how the different calibration methods perform for the same scenario. The MASE allows for us to compare across scenarios, to understand if methods perform differently for varying levels of isolation and vaccination in the population. We previously discussed and presented the formula for MASE in Section 2.3.1.

The formula for the RRMSE is:

(16) $$\frac{1}{\bar{Y}}\sqrt{\overset{n}{\underset{i=1}{\sum }}\frac{{\left(Y_{t}-F_{t}\right)}^{2}}{n}}$$

where Y is the ground truth data, F is the forecasted or predicted data from the calibrations, n is the number of data points, and $\bar{Y}$ is the average ground truth data (Chen et al., 2016). The RRMSE is the root mean squared error (RMSE) divided by the mean of the observed values. The RRMSE for each scenario is calculated the same way as the MAE is: first finding the RRMSE for each of the 100 Nelder-Mead samples and 4000 HMC samples and then taking the mean across all RRMSEs. One of the main advantages of the relative measures of error are there interpretability. A value close to 0 indicates a good fit, while a value of the RRMSE above 1 would indicate that the model may not be performing well. However, the RRMSE is more susceptible to outliers than the MAE (Hyndman & Koehler, 2006). Looking at all three values of accuracy should provide a comprehensive overview of the accuracy of the two calibration methods.

## 3 Results

### 3.1 Model performance

The following sections explore the first aim of our study: to understand what methods of model calibration are most accurate compared to the ground truth case data when all information is known. For the two calibration methods selected, Nelder-Mead and HMC, we compare the forecasted values for new cases per day from the calibration to the ground truth data and assess model accuracy using MASE and MAE.

#### 3.1.1 Nelder-Mead

Before looking at the accuracy of the Nelder-Mead models it is important to assess the performance of the calibration. One way to do this is to examine the convergence of the model. The Nelder-Mead method can have difficulty converging especially at local minimum (Maia et al., 2017). To assess the convergence of the Nelder-Mead method we look at the number of times the Nelder-Mead method reaches the maximum number of iterations (this is set to 700). Although this will not tell us if the Nelder-Mead method has stopped on a local minimum, it will provide information about the convergence of the method. Table 2 presents the results for the Nelder-Mead convergence. For all scenarios we see that the model performs relatively well with high convergence rates.

**Table 2:** Percent of iterations divergent in the Nelder-Mead Calibration.

Scenario	Percent Divergent
S1_I0_V0	0.02
S2_I50_V0	0.02
S3_I100_V0	0.03
S4_I0_V50	0.00
S5_I50_V50	0.00
S6_I100_V50	0.05
S7_I0_V80	0.05
S8_I50_V80	0.05
S9_I100_V80	0.12

Table 3 shows the MASE, MAE and RRMSE value for each scenario. Across all scenarios, the MAE values are small compared to the number of cases in the scenario. As MAE is relative to scale, this suggests that the calibrated models are performing well. In all scenarios except for Scenario 3 (0 % vaccination and 100 % isolation), the MASE value is less than 1. This suggests that the calibrated model might be performing better than the naive model in most cases. The RRMSE is less than 1 for all scenarios, suggesting the model is performing well for most scenarios.

**Table 3:** Accuracy measures for the Nelder-Mead and HMC Calibration.

Scenario	Nelder-Mead			HMC		
	MASE	MAE	RRMSE	MASE	MAE	RRMSE
S1_I0_V0	0.77	29.11	0.61	1.45	32.37	0.78
S2_I50_V0	0.71	31.74	0.88	1.61	28.42	0.91
S3_I100_V0	1.09	24.50	0.46	1.88	20.64	0.46
S4_I0_V50	0.66	9.46	0.47	1.16	11.81	0.72
S5_I50_V50	0.67	7.24	0.45	1.11	8.88	0.69
S6_I100_V50	0.68	7.00	0.21	1.2	8.63	0.31
S7_I0_V80	0.67	2.33	0.72	1.97	4.79	1.69
S8_I50_V80	0.71	1.21	0.72	1.4	2	1.11
S9_I100_V80	0.71	1.25	0.42	1.28	1.63	0.62

#### 3.1.2 HMC

To assess the performance of the calibrations we look at the divergence of the HMC iterations. An iteration is divergent when there is too much of a departure between the trajectory simulated from the calibration and the true trajectory. Divergence suggests the inference from the divergent iteration might be biased (Margossian et al., 2020). In Stan, the divergence from the true trajectory is determined by comparing the Hamiltonian value with initial value, the default threshold for divergence is a factor of 10^3^ Stan Development Team (2024). Table 4 shows the percent of iterations for each scenario that are divergent. From the table we can see that the percent of iterations that are divergent for each scenario are small, with only one scenario diverging more than 2 % of the iterations, and even there the percent of iterations that are divergent are 2.1 %. This suggests that the calibration is performing relatively well. Further diagnostic metrics are included in the Supplementary materials.

**Table 4:** Percent of iterations divergent in the HMC Calibration.

Scenario	Percent Divergent
S1_I0_V0	1.30
S2_I50_V0	1.53
S3_I100_V0	1.74
S4_I0_V50	2.10
S5_I50_V50	1.70
S6_I100_V50	0.62
S7_I0_V80	0.82
S8_I50_V80	0.22
S9_I100_V80	0.30

Table 3 presents the accuracy measures for the HMC calibration. Looking at the MAE values for the HMC calibrations, they are small relative to the number of cases in the scenarios. HMC performs better for the models with 0 % and 50 % vaccination rates, with a relatively small MAE for these scenarios. However, HMC does not seem to perform as well for the scenarios with 80 % vaccination, with a higher MAE compared to the outbreak sizes. Due to the high vaccination rates in these scenarios the outbreak sizes are small, which might suggest that the HMC method does not work as well when there are small case numbers. The average MASE for all scenarios is above 1 suggesting that the HMC calibrated model might not perform better than the naive model. Looking at the RRMSE, we see similar to the MAE that while the HMC performs well for the first six scenarios, the RRMSE is greater than 1 for two of the scenarios with 80 % vaccination suggesting poor model performance for these scenarios.

#### 3.1.3 Comparison

**Fig. 5:**
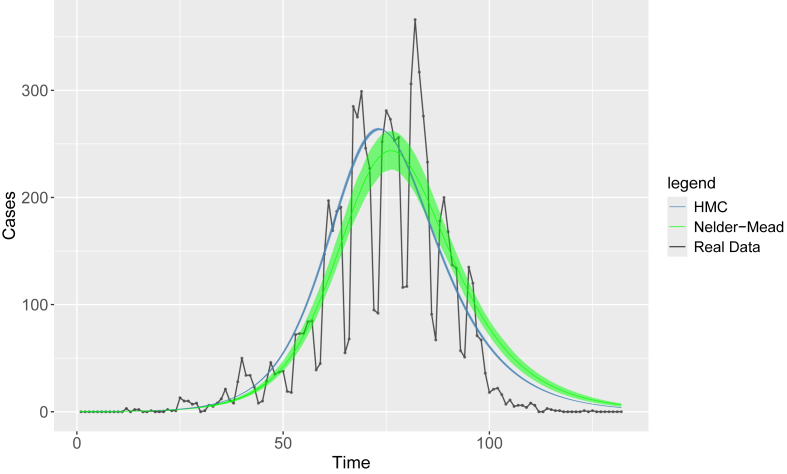
Example of calibration for a single time series of the synthetic data when there is 0 % vaccination and 0 % isolation. The dots are the real data, blue lines HMC model and 75 % credibility interval and green lines are Nelder-Mead model and 75 % credibility interval.

Fig. 5 shows the synthetic data for the new cases per day for one run of Scenario 1 of the agent-based model (isolation 0 % and vaccination rate 0 %), the predicted cases per day and the 75 % credible intervals from the Nelder-Mead and HMC calibrations. It is important to note that this figure is for visualization of the calibrations and are only one run of the 50 runs for the scenario. As such conclusions should not be drawn from this figure. However, the plot provides a depiction of the fits, showing the plausible ranges of parameters from the fits and how the fits compare to the synthetic data. We can see for this fit the Nelder-Mead (green line) has a wider distribution of plausible values compared to the HMC (blue lines) and the HMC predicts a slightly earlier peak compared to the Nelder-Mead.

Interestingly, when looking at the accuracy measures, Nelder-Mead performs slightly better for the majority of the scenarios. Although in some scenarios there is little difference between the performance of the methods.

### 3.2 Parameter values

The following sections explore the second aim of our study: to understand how changing contact patterns can impact the effective parameters of an infectious disease. We look at the distributions of the calibrated parameters for both of Nelder-Mead and HMC methods and assess how the parameters and distributions change as the isolation and vaccination rates of the system change.

#### 3.2.1 Nelder-Mead

The Nelder-Mead calibration quantiles for infectious period can be found in Table 5. The infectious period is found by taking the inverse of the *γ* parameter. Interestingly, the scenarios with the closest medians to the ground truth infectious period are Scenarios 7 and 8 both with 80 % vaccination rate. These scenarios have isolation rates of 0 % and 50 % respectively. For all three vaccination rates, we see a reduction in the infectious period as the isolation rate increases. The reduction is most pronounced in the scenarios with 80 % vaccination rate and least pronounced with 50 % vaccination. However, we do not see a decrease in infectious period in the scenarios with 0 % vaccination until the third scenario when agents isolate 100 % of their infectious period. While the quantiles have a smaller range for the scenarios with 50 % vaccination rate, the range of other scenarios is wider. With some having the range the entire parameter space. Interestingly, we can see that for the majority of scenarios the highest median infectious periods are for the 80 % vaccination rates. This highlights the adaptation of the parameters for the SEIR process model. As the data is generated from a model with heterogeneous mixing while the process model assumes homogeneous mixing the model parameters have to adapt to account for this. One way to adapt and account for a higher case count that might occur with a heterogeneous mixing model, is to increase the infectious period allowing for more contacts and potential infections in the homogeneous process model.

**Table 5:** Quantiles for the distribution of infectious period for the Nelder-Mead and HMC methods of calibration.

Scenario	2.50 %	5 %	25 %	median	75 %	95 %	97.50 %
Nelder-Mead							
S1_I0_V0	1.00	1.00	4.00	6.11	9.31	10.00	10.00
S2_I50_V0	1.01	1.73	4.50	6.05	7.93	10.00	10.00
S3_I100_V0	1.00	1.00	1.00	1.00	1.00	1.42	8.27
S4_I0_V50	1.68	1.69	1.72	1.76	2.05	2.85	3.40
S5_I50_V50	1.45	1.46	1.48	1.53	1.62	2.21	2.57
S6_I100_V50	1.35	1.36	1.37	1.38	1.46	1.63	1.85
S7_I0_V80	7.52	7.67	8.50	9.00	9.62	10.00	10.00
S8_I50_V80	3.48	3.66	4.42	6.42	7.64	9.75	9.86
S9_I100_V80	2.22	2.22	2.35	2.52	3.11	4.79	7.57

HMC							
S1_I0_V0	9.63	9.69	9.85	9.92	9.97	9.99	10.00
S2_I50_V0	9.71	9.76	9.88	9.94	9.98	10.00	10.00
S3_I100_V0	1.00	1.00	1.01	1.03	1.04	1.06	1.07
S4_I0_V50	1.83	1.83	1.85	1.87	1.92	1.96	1.97
S5_I50_V50	1.57	1.57	1.58	1.60	1.63	1.67	1.67
S6_I100_V50	1.47	1.47	1.48	1.50	1.54	1.56	1.57
S7_I0_V80	8.21	8.28	9.68	9.99	10.00	10.00	10.00
S8_I50_V80	4.86	4.98	5.38	5.68	5.97	7.41	8.43
S9_I100_V80	2.50	2.54	2.57	2.60	2.67	2.72	2.73

Table 6 provides the quantiles for each of the scenarios for *β*. From the table we can see that there is a range of *β* values across the scenarios. The lowest transmission rates are for Scenarios 1 and 2, where there is no vaccination, and for Scenarios 7 and 8, where there is 80 % vaccination. In these scenarios there is either no isolation or 50 % isolation. These four scenarios have median values of *β* between 0.61 and 0.99 while the other scenarios have median values for *β* between 2.07 and 2.50. The scenarios with low values for *β* correspond with the scenarios with high values or infectious period in Table 5. As agents appear to have a longer effective infectious period in these scenarios this may translate to having less effective transmission. Because agents have contact networks there is a set number of other agents that they have a high probability of coming into contact with where transmission events might occur. If they have six days to infect their contacts versus one day their effective transmission rate will be lower over the six days. Across the majority of scenarios, with the exception of Scenario 7, we see a wide range in the *β* values between the 2.5 % and 97.5 % quantiles.

**Table 6:** Quantiles for the distribution of *β* for the Nelder-Mead and HMC methods of calibration.

Scenario	2.50 %	5 %	25 %	median	75 %	95 %	97.50 %
Nelder-Mead							
S1_I0_V0	0.54	0.54	0.58	0.70	0.92	2.50	2.50
S2_I50_V0	0.44	0.45	0.52	0.61	0.74	1.31	1.53
S3_I100_V0	0.28	1.05	1.49	2.16	2.17	2.18	2.19
S4_I0_V50	1.45	1.66	2.06	2.44	2.50	2.50	2.50
S5_I50_V50	1.49	1.77	2.31	2.50	2.50	2.50	2.50
S6_I100_V50	1.93	2.12	2.48	2.50	2.50	2.50	2.50
S7_I0_V80	0.74	0.75	0.77	0.81	0.85	0.92	0.93
S8_I50_V80	0.70	0.75	0.84	0.99	1.25	1.64	1.69
S9_I100_V80	0.79	1.16	1.77	2.07	2.34	2.49	2.49

HMC							
S1_I0_V0	0.59	0.59	0.59	0.59	0.59	0.59	0.60
S2_I50_V0	0.52	0.52	0.52	0.52	0.52	0.53	0.53
S3_I100_V0	2.35	2.36	2.40	2.42	2.44	2.45	2.45
S4_I0_V50	2.35	2.37	2.42	2.46	2.48	2.50	2.50
S5_I50_V50	2.37	2.39	2.44	2.47	2.49	2.50	2.50
S6_I100_V50	2.42	2.43	2.46	2.48	2.49	2.50	2.50
S7_I0_V80	0.93	0.95	2.05	2.45	2.49	2.50	2.50
S8_I50_V80	0.95	1.02	1.24	1.68	2.25	2.50	2.50
S9_I100_V80	2.33	2.34	2.41	2.47	2.49	2.50	2.50

The *R*_*c*_ distributions for the scenarios calibrated using Nelder-Mead along with the reference *R*_*c*_ for each of the nine scenarios are in Table 7. From the table we can see that the highest *R*_*c*_ values are for the scenarios with 80 % vaccination, while the *R*_*c*_ values for the scenarios with no vaccination and 50 % vaccination are more similar. The high values for *R*_*c*_ in the 80 % vaccination scenarios are likely due to the difference in assumed contacts between the process model and the agent-based model. The process model assumes homogeneous mixing while in the agent-based model agents will mix heterogeneously among their contact networks. The contact networks might lead to a greater overall outbreak size due to the virus spreading within and between networks. As infectious agents will have more contact with those in their network they are more likely to pass on the infection compared to a random mixing scenario. Therefore, in order for the process model to capture the higher numbers of cases from the agent-based model, the model assumes that the disease is more infectious resulting in higher *R*_*c*_ values. For each set of vaccination scenarios, the highest *R*_*c*_ is for the scenario with no isolation and the lowest for the scenarios with 100 % isolation. All the values for *R*_*c*_ are below the ground truth *R*_0_, 12. However, while the calibrated *R*_*c*_ for scenarios with vaccination rates of 0 % and 50 % are below the ground truth *R*_*c*_, the calibrated *R*_*c*_ for the scenarios with vaccination rates of 80 % are greater than the ground truth *R*_*c*_.

**Table 7:** Quantiles for the distribution of the Control Reproduction Number for the Nelder-Mead and HMC methods of calibration.

Scenario	*R*_*c*_	2.50 %	5 %	25 %	median	75 %	95 %	97.50 %
Nelder-Mead								
S1_I0_V0	12	2.50	2.50	3.40	4.11	5.03	5.40	5.58
S2_I50_V0	12	1.97	2.50	3.07	3.44	3.92	4.44	4.61
S3_I100_V0	12	1.43	144	1.53	2.21	2.23	2.24	2.26
S4_I0_V50	6.18	4.20	4.21	4.26	4.29	4.42	4.64	4.99
S5_I50_V50	6.18	3.60	3.62	3.66	3.69	3.72	3.83	4.02
S6_I100_V50	6.18	3.38	3.38	3.41	3.44	3.47	3.51	3.60
S7_I0_V80	2.69	6.90	6.93	7.05	7.13	7.16	7.28	7.35
S8_I50_V80	2.69	5.92	5.94	6.00	6.12	6.22	6.49	6.56
S9_I100_V80	2.69	5.45	5.46	5.49	5.53	5.56	5.65	5.77

HMC								
S1_I0_V0	12	5.73	5.75	5.82	5.84	5.86	5.88	5.89
S2_I50_V0	12	5.04	5.05	5.09	5.11	5.13	5.15	5.15
S3_I100_V0	12	2.44	2.44	2.45	2.45	2.46	2.46	2.47
S4_I0_V50	6.18	4.48	4.49	4.51	4.52	4.54	4.62	4.64
S5_I50_V50	6.18	3.89	3.90	3.92	3.93	3.95	3.96	3.97
S6_I100_V50	6.18	3.66	3.66	3.68	3.69	3.70	3.71	3.72
S7_I0_V80	2.69	7.32	7.36	7.51	16.05	24.94	24.99	24.99
S8_I50_V80	2.69	6.30	6.31	6.33	6.57	6.85	7.15	7.33
S9_I100_V80	2.69	5.82	5.84	6.16	6.18	6.20	6.23	6.23

**Fig. 6:**
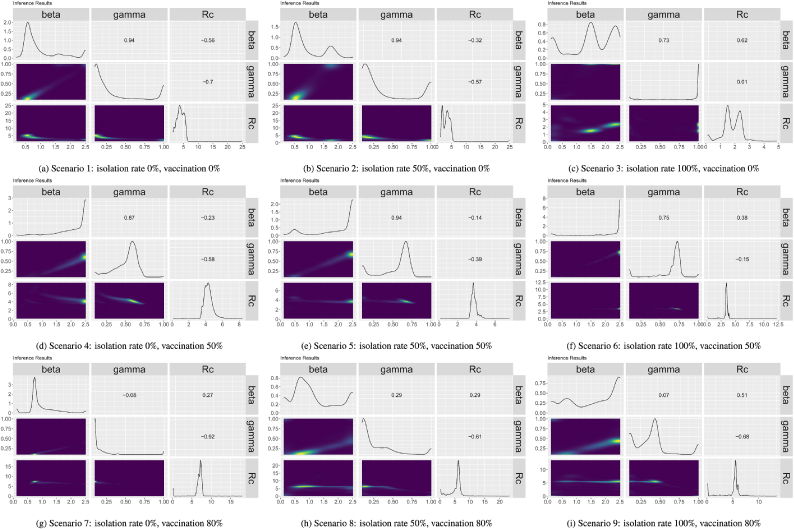
Pairwise plots of the distribution of *β*, *γ* and *R*_*c*_ from the Nelder-Mead calibration of different scenarios.

Fig. 6 shows the pairwise plots for the distributions of *β*, *γ* and *R*_*c*_ across all ground truths for each of the scenarios. The diagonals of the plots show the distributions for each parameter, the top right half of the figures show the correlations and the bottom right the joint distributions. In the joint distributions, the more yellow the plot is the higher the probability density at those points. Looking at the distributions for each single parameter, the majority have multiple peaks in the parameter distributions suggesting that there might be multiple combinations of parameters that will lead to an optimized model. This is further seen in the joint distribution plots where there tends to be a high density yellow area on the plot with a long tail. For most of the scenarios, there is a high correlation between *β* and *γ*. Six of the nine scenarios have correlations of 0.75 or higher. The correlations of the other three scenarios range from 0.29 to −0.08: isolation rate 100 % and vaccination 80 % correlation 0.07), isolation rate 50 % and vaccination 80 % (correlation 0.29), and isolation rate 0 % and vaccination 80 % (correlation −0.08). The correlations between *γ* and *R*_*c*_ for all but one scenarios are negative. There does not seem to be a pattern or similarities in the correlation between *β* and *R*_*c*_ across scenarios.

#### 3.2.2 HMC

The quantiles for the infectious periods for each scenario from the HMC calibration can be found in Table 5. The infectious periods are calculated by taking the inverse of the *γ* parameter. There are some patterns in the data that are worth noting, there appears to be a reduction in the estimated infectious period as the isolation period increases. In these instances, the calibration is likely capturing the effective infectious period. However, the decrease in infectious period is most prominent in the estimated values for the scenario with 80 % vaccination and negligible in the scenarios with 50 % vaccination, suggesting that the estimated parameter for infectious period is not only capturing the reduction in time that an individual might be infectious but also potentially interactions with other factors within the model. It can be noted that the infectious period for Scenarios 1, 2 and 7 are close to the ground truth infectious period that was used in the agent-based model (on average 8 days). Scenarios 1 and 2 are both scenarios with 0 % vaccination. This suggests that when there is no vaccination and when individuals isolate for up to half of their infectious period, using HMC to calibrate the model to data might capture the real infectious period. Scenario 7 suggests that when there is vaccination, the real infectious period may still be captured in scenarios where no one isolates while infectious. The distribution of infectious periods across the 50 ground truths is small, less than one day for seven of the nine scenarios. The two scenarios that have wider distributions are both scenarios with 80 % vaccination. Similar to the Nelder-Mead calibration the infectious periods tend to be larger in the 80 % vaccination scenarios suggesting the parameters are adjusting for the changes in model assumptions between the data generating model and the process model.

The quantiles for the *β* parameter, or the transmission parameter, can be found in Table 6. Its notable that the distributions of *β* are similar across the majority of the scenarios, with a median of approximately 2.4 Scenario 8, 80 % vaccination and 50 % isolation has a lower median compared to most scenarios (1.68), however, there is wide distribution of and a 97.5 % quantile interval between 0.95 and 2.5. Scenarios 1 and 2 have *β* values less than 1, these are the two scenarios with 0 % vaccination that have an infectious period close to the ground truth infectious period.

Table 7 shows the quantiles for *R*_*c*_ derived from the *β* and *γ* parameters as well as the ground truth *R*_*c*_ for each scenario. From the table we can see that the scenarios with the highest *R*_*c*_ are those with 80 % vaccination rates. In fact the scenario with 80 % vaccination and 0 % isolation has an *R*_*c*_ closest to that of the *R*_0_ from the ground truth agent-based model and a plausible value for the *R*_0_ of measles (12–18). However, this scenario has the largest range of *R*_*c*_ values across the 97.5 % quanitles of all the scenarios ranging from 7.32 to 24.99. The scenarios with 80 % vaccination rates are also the only scenarios with a calibrated *R*_*c*_ that is higher than the ground truth *R*_*c*_. Interestingly, while scenarios 3 through 7 and 9 all have similar *β* (infection rate) values, the *R*_*c*_ for these scenarios vary between 2.45 and 6.18, showing the influence of both vaccination rates and isolation rates on the *β* values.

**Fig. 7:**
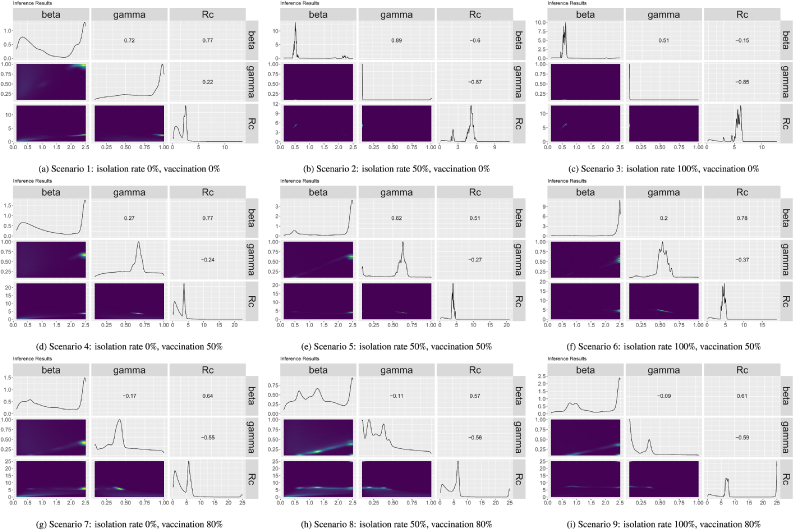
Pairwise plots of the distribution of *β*, *γ* and *R*_*c*_ from the HMC calibration of different scenarios.

Fig. 7 shows pairwise plots for the distributions of *β*, *γ*, and *R*_*c*_ across all ground truths for each of the nine scenarios. Looking at the single parameter distributions along the diagonals of the pairwise plots, although there is some noise three of the nine scenarios appear to have a single peak in the distribution of parameters (isolation rate 0 %, vaccination rate 0 %, isolation rate 50 %, vaccination rate 0 % and isolation rate 0 %, vaccination rate 50 %) while the other scenarios have two peaks or more peaks in some of their parameters. This can be seen also in the joint density plots where the scenarios with single peaks appear to have one region with higher probability density while the other scenarios appear to either have either a joint probability distribution with a long tail or multiple high probability areas on the plot. The correlations between *β* and *γ* appear to vary by the vaccination rates in the scenarios. For the scenarios 0 % vaccination *β* and *γ* have high positive correlations (between 0.51 and 0.89); for the scenarios with 50 % vaccination *β* and *γ* have lower but still positive correlations (between 0.2 and 0.69); and for the scenarios with 80 % vaccination *β* and *γ* have low negative correlations (between −0.09 and −0.17). Looking at the correlations between *β* and *R*_*c*_ and *γ* and *R*_*c*_, for all the scenarios with 50 % and 80 % vaccination there is a positive correlation between *β* and *R*_*c*_ and a negative correlation between *γ* and *R*_*c*_. In the scenarios with 0 % vaccination and either 0 % isolation or 50 % isolation the correlations between *β* and *R*_*c*_ and *γ* and *R*_*c*_ are both negative while the correlations are both positive for the scenario with 0 % vaccination and 100 % isolation.

#### 3.2.3 Comparison

**Fig. 8:**
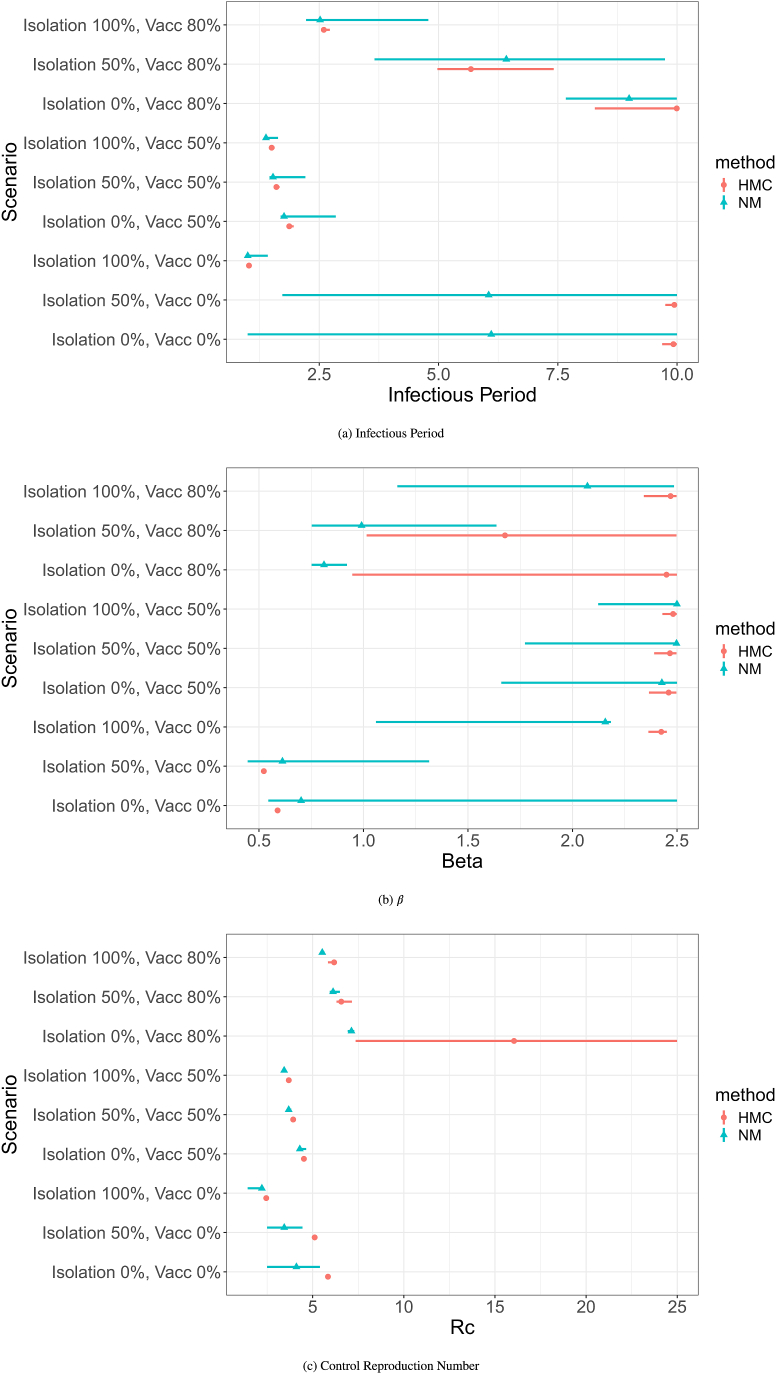
95 % quantiles for the calibrated model parameters.

Fig. 8 compares the quantile distributions for the HMC and Nelder-Mead calibrations for the infectious period, *β*, and *R*_*c*_ by scenario. From the plot it appears that for the majority of Scenarios, the Nelder-Mead calibrations have wider quantile distributions. The only exception are for the infectious period and *R*_*c*_ for Scenarios 7 and 8 (80 % vaccination and isolation at 0 % and 50 % respectively) where the HMC results in a wider distribution. Although, it appears that the *R*_*c*_ intervals are comparatively much smaller compared to the infectious period and *β*, this is likely due to the overall wider range of values for *R*_*c*_ resulting in a longer x-axis making the intervals appear smaller.

Looking at the infectious period intervals, Fig. 8a, the changes in size of infectious period as the contact pattens of the infectious agent changes is clear. The pattern is particularly clear for the especially the case for the 80 % vaccination rate scenarios with HMC, but a decrease can also be seen in the 0 % vaccination scenarios in the HMC calibration between the 50 % isolation and 100 % isolation. Although *β* also changes as the contact patterns of the agents change, the pattern is not as clear as it can be seen for infectious period.

Additionally, when comparing the median values between the Nelder-Mead calibrations and the HMC calibrations, it appears that there is a greater difference in the medians for the infectious period and *β* across scenarios than for *R*_*c*_. This suggests that both methods might be finding the overall reproduction number of the system, but through different possible sets of parameters.

## 4 Conclusion

### 4.1 Summary and discussion

In this work an agent-based model was used to generate different scenarios for a measles outbreak in a town in Ireland, altering the parameters for both the vaccination rates in the town and for the rates agents will isolate when infectious. The ground truth scenario data was used to calibrate a deterministic compartmental process model using both Nelder-Mead, an optimization calibration technique, and HMC, a Bayesian calibration technique. Both the performance of the calibrated models and the values of the estimated model parameters were evaluated. Interestingly when looking at accuracy measures, the Nelder-Mead algorithm performs slightly better than HMC. When comparing the MAE values for the Nelder-Mead and HMC calibrations, we mostly do not see a difference in performance between the methods. The one possible exception is for the scenarios with 80 % vaccination where the Nelder-Mead appears to perform slightly better than the HMC. This could be due to the smaller number of cases in the scenarios with 80 % vaccination rates. The estimated parameter intervals produced by HMC for the 80 % vaccination scenarios are larger than those for other scenarios suggesting HMC has a harder time fitting to these scenarios with low case numbers. However, when looking at the convergence of the two methods, the HMC converged more often compared to the Nelder-Mead method. This is a known drawback of the Nelder-Mead method. Comparing the parameters from the calibration methods, the HMC method seems to match more closely with the ground truth parameters especially in the scenarios with no vaccination and no isolation, but also in the scenarios with 80 % vaccination and no isolation. The *no isolation* and the *no vaccination* scenarios are those we would expect to be closest to the ground truth parameters as the output of the model is not influenced by either a larger number of vaccinated individuals reducing the susceptible population or a change in the contact patterns of the infectious individuals when they isolate. Additionally, we find that the distributions of the HMC parameters are narrower compared to the Nelder-Mead parameters. These results highlight that HMC might perform slightly better than Nelder-Mead for calibration to infectious disease data. Other work has found similar results to our analysis. Mumtaz (2024) compared HMC and a bootstrapping method using Powell's method and found that when calibrating to a small number of parameters, 2 or 3, bootstrapping performed slightly better than HMC. Andrade and Duggan (2020) find that NM and HMC are both robust methods to estimate *R*_0_, but HMC produces the most accurate fits. Mumtaz (2024) also find that the HMC produced narrower credible intervals compared to bootstrapping.

### 4.2 Insights

The analysis of the change in model parameters as the contact patterns and number of susceptible agents in the population changes reveals valuable insights. We show that the effective infectious period determined through calibration and to a lesser extent the *β* parameter are sensitive to the isolation rates and the vaccination rates of the system. We find that for each vaccination rate, as the isolation rate increases the infectious period decreases. When there is full susceptibility in the population in some scenarios the effective infectious period tends to the actual infectious period. However, this is not the case for the 50 % vaccination scenarios suggesting that there are interactions between the two parameters that might not be captured in the process model formulation. The sensitivity of the calibration to the change in isolation rates and vaccination rates shows that when there is a change in the data generating process, in this case the agent-based model, this change is reflected in difference in the parameters that define the process SVEIR model. Translating this to a real world potential scenario, if a SEIR compartmental model is being calibrated to real data we might expect the estimated infectious period to increase in a situation where contacts are increased, for example around the holiday season.

What is, however, particularly interesting is that while *β* and the infectious period change between scenarios, they tend to change in tandem, e.g. a higher *β* tends to pair with a lower infectious period. Although there are some variations, this results in more similar *R*_*c*_ values across all scenarios and suggests that the *R*_*c*_ might be a driver of the dynamics of the infectious disease spread in the agent-based model and as such the calibration methods preserve *R*_*c*_ which is derived from ratios of *β* and the infectious period.

Understanding how the calibrated parameters change as certain characteristics of the system that generates the data change, is important in understanding how to interpret the parameters of a similarly calibrated model when the exact parameters of the system are not known. For example, when calibrating a model to real outbreak data instead of synthetic data. Our results show that if there is no vaccination or prior immunity in the system and low levels of isolation, 50 % or below or high vaccination rates but no isolation, the effective infectious period from the calibrated model will likely be similar to the real infectious period. However, if there is a higher isolation rate or a higher vaccination rate, then the effective infectious period appears to be a combination of both the actual infectious period and the contact structure of the agents in the model. This would suggest that it is important to calibrate a model to new data if either the isolation rate or vaccination rates of a system change. For example, if a model was calibrated to measles data before the introduction of a vaccine, the effective parameters from that model will likely not be useful in understanding a current measles outbreak where there is a large percent of the population either vaccinated or who have prior immunity from infection. Additionally, if there is situation where the parameters from a calibrated model are being used to understand an infectious disease where the actual infectious period is unknown, it is important to look at the behaviours of the individuals in the population where the data was collected. If there is a high level of isolation or immunity in the population it should be included in the interpretation of the parameters that the real infectious period is likely higher than the effective infectious period found through calibration.

### 4.3 Limitations and future work

There are a number of limitations to our study. In using the agent-based model to create synthetic data we made a number of assumptions in the model that might not be highly realistic. Vaccination rates in the model are unrealistic for measles, all of our scenarios included vaccination rates that were lower than the actual vaccination rates in Ireland. Additionally, the vaccination rates were evenly applied across the population whereas in reality vaccinations are likely to vary by age and other factors such as socioeconomic status (Doherty et al., 2014; HPSC, 2025). In Ireland, measles is a notifiable disease and as such for any suspected cases of measles in Ireland, it is required that there is immediate preliminary notification by telephone to a Medical Officer of Health (HPSC, 2024). Therefore, by selecting measles as the disease being modeled it allowed us to the full data from the agent-based model and not consider reported vs unreported cases. The results of our analysis might be different if a disease such as flu or COVID-19 where asymptomatic cases and under-reporting are important factors in the dynamics of the disease (Kalachev et al., 2024). Future work could look at not only the analysis of more realistic vaccination rates but could also look at other diseases such as influenza or COVID-19. Also, while the agent-based model a normal distribution is used to determine the incubation and infectious periods for each infected agent, our SEIR model assumes an exponential distribution for these parameters (Hong et al., 2024). A difference in the assumed distribution of the parameters could result in bias in the inferred parameters, however, in reality, incubation and infectious periods are not exponentially distributed (Wearing et al., 2005). Thus, calibrating an SEIR model to real-world data will result in a similar bias.

In addition, in our calibration procedures we restricted the range of the parameters based on our knowledge of possible values used in the agent-based model. We did not calibrate to certain parameters such as the incubation period that we did not think would be altered by changing the isolation rates or contact patterns. However, these restrictions require some knowledge of the system already and might not be possible in a real world scenario. Work could be done to analyse how different restrictions and including different parameters in calibration might impact the results. In particular as the performance of the Nelder-Mead method is known to rely on the initial conditions, important future work will be in looking at how selecting from a range of possible values for the initial conditions will impact the results.

Furthermore, the process model we use only considers one age group while the agent-based model allows for different mixing by age. Comparing the agent-based model data to a more robust process model such as that used in (Andrade & Duggan, 2020) with different age cohorts might impact our results as well. Other compartments that might better capture the social isolation or differences in social mixing could also be included in the process model. However, it is important to understand how the parameters in even a simple SEIR model change as behaviours change because we do not always know the exact causes of changes in behaviours to add compartments to the model. While our data is being generating by stochastic process, the process model is deterministic. Other work has been done with stochastic process models (Andrade & Duggan, 2022), and it might improve model calibration and interpretation to use a stochastic process model to model a stochastic system. In future work, we aim to extend the process model through adding stochasticity. Additionally, in this work we focus on parameter estimation and not forecasting. However, in many instances process models are calibrated to data in order to predict or forecast cases. Future work will also focus on looking at the forecasting performance of the model using different calibration methods.

## CRediT authorship contribution statement

**Elizabeth Hunter:** Writing – original draft, Methodology, Formal analysis, Conceptualization. **Jim Duggan:** Writing – review & editing, Supervision, Methodology, Funding acquisition, Conceptualization.

## Declaration of competing interest

The authors declare that they have no known competing financial interests or personal relationships that could have appeared to influence the work reported in this paper.

## Footnotes

1 A random number is drawn from a uniform distribution between 0 and 1 and if that random number is less than the infection probability then the susceptible agent will become exposed.
