# Supplementary material for: A multi-method study evaluating the inference of compartmental model parameters from a generative agent-based model
Source: Infect Dis Model. 2025 Oct 16;11(1):218–40. doi: 10.1016/j.idm.2025.10.002 (PMC12593580; doi:10.1016/j.idm.2025.10.002)
Supplement: Multimedia component 1 [file mmc1.pdf]

Supplementary Material

Contents

1 Bootstrapping **1**

2 HMC Diagnostics **1**

3 Single Variable Calibrations **4**

3.1 β 4

3.1.1 Nelder-Mead 4

3.1.2 HMC 5

3.2 γ 7

3.2.1 Nelder-Mead 7

3.2.2 HMC 9

1 Bootstrapping

For a better understanding and visualization of the bootstrapping used for the Nelder-Mead calibration, the Figures A.1, A.2, A.3 and A.4 show the 100 data series generated from a single run of the synthetic data set with 0% isolation and 0% vaccination.

2 HMC Diagnostics

The following section provides additional HMC diagnostic information. Stan provides a set of diagnostic tools that can be used to understand the reliability of the inference. One measure that is used is $\hat{R}$, which is the ratio of the total variance to the within chain variance. An $\hat{R}$ close to 1 suggests that the inference is reliable grinsztajn2021bayesian. Another reliability measure that we can find through Stan is the effective sample size. The effective sample size is an estimate of the number of independent draws from the posterior distribution that are represented in the output CHATZILENA2019100367. If the effective sample size is large enough, 5 times twice the number of chains used in the inference, the inference can be thought of as reliable gelman2013bayesian. Stan provides the effective sample size for the bulk or the majority of the posterior distribution and the tail of the distribution separately. Table A.1 shows the $\hat{R}$

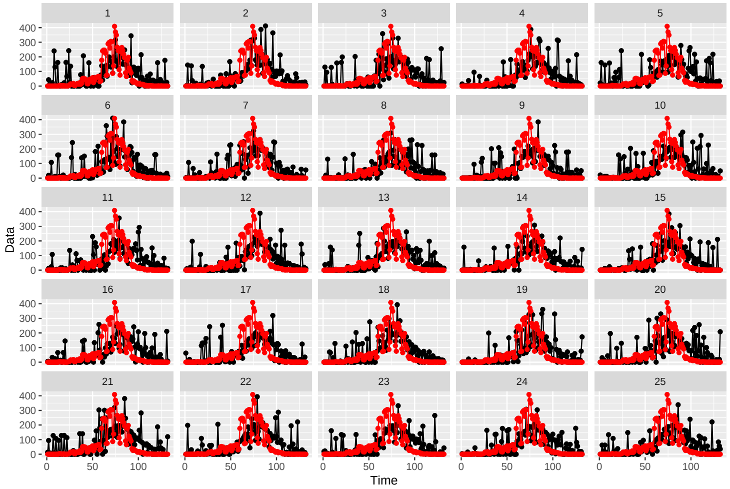

Figure A.1: Data series generated from bootstrapping for the Nelder-Mead Calibration. The black points are the bootstrap data series while the red points represent the single run of the synthetic data.

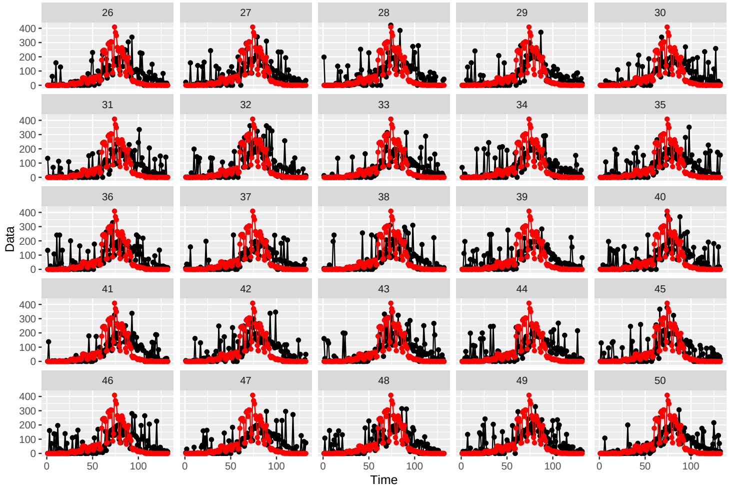

Figure A.2: Data series generated from bootstrapping for the Nelder-Mead Calibration. The black points are the bootstrap data series while the red points represent the single run of the synthetic data.

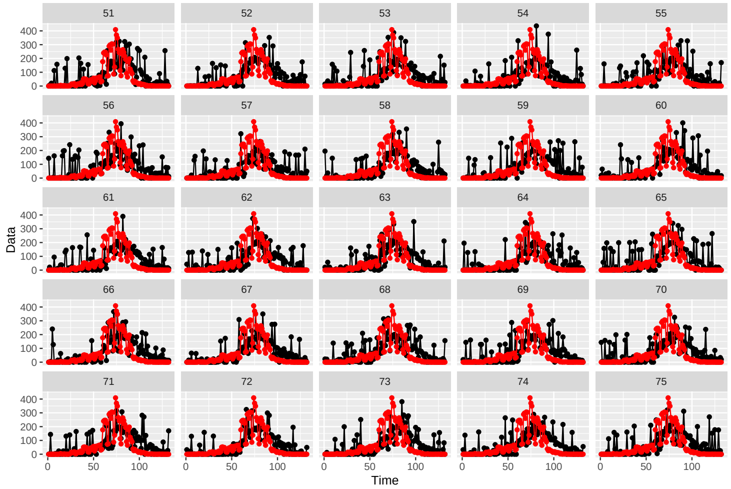

Figure A.3: Data series generated from bootstrapping for the Nelder-Mead Calibration. The black points are the bootstrap data series while the red points represent the single run of the synthetic data.

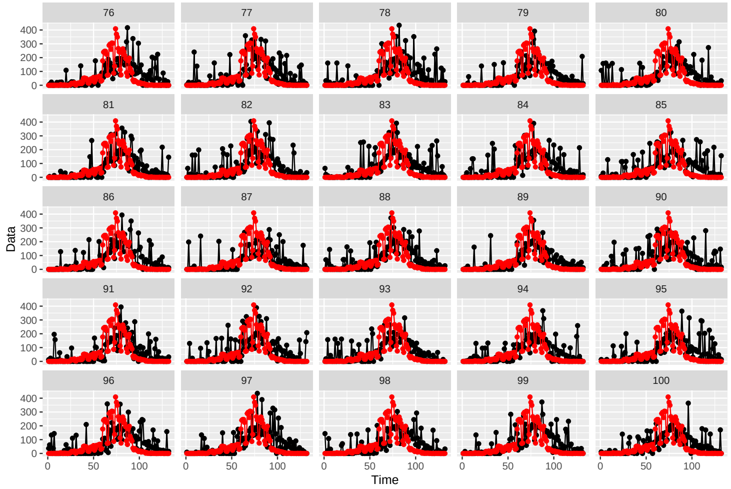

Figure A.4: Data series generated from bootstrapping for the Nelder-Mead Calibration. The black points are the bootstrap data series while the red points represent the single run of the synthetic data.

Table A.1: Diagnostic measures for the HMC calibration

	β			γ		
Scenario	$\hat{R}$	ESS bulk	ESS tail	$\hat{R}$	ESS bulk	ESS tail
S1_I0_V0	1.03	682.27	463.10	1.03	602.90	520.66
S2_I50_V0	1.04	601.84	379.05	1.03	552.49	457.45
S3_I100_V0	1.03	1288.29	994.29	1.03	1349.41	1112.31
S4_I0_V50	1.08	385.66	330.55	1.08	356.15	304.85
S5_I50_V50	1.07	527.58	414.59	1.07	562.97	438.41
S6_I100_V50	1.03	1711.91	1271.17	1.03	1768.37	1339.22
S7_I0_V80	1.23	721.22	582.73	1.24	713.90	587.91
S8_I50_V80	1.11	1290.50	1029.26	1.11	1244.21	1006.79
S9_I100_V80	1.03	1720.07	1266.94	1.03	1736.27	1304.46

values and the effective sample size bulk and tail for the β and γ estimates for each of the scenarios. From the tables we can see that $\hat{R}$ values are close to 1 and the effective sample sizes appear large enough for each of the scenarios. Thus suggesting a reliable inference.

3 Single Variable Calibrations

In the main text we use the Nelder-Mead and Hamiltonian Monte Carlo (HMC) calibration methods to find plausible values for two parameters of our deterministic process model. The two parameters, β (the transmission rate) and γ (the inverse of the infectious period), are the parameters in the model that could be influenced by the change in behaviours and interactions of individuals. However, these two parameters are highly correlated, as noted in the text when β increases γ decreases and vice versa. Here we present the results of single variable calibration where we run the calibration methods holding γ constant at the reference value of $\frac{1}{8}$ to find β and holding β constant at the reference value of 1.5 to find γ. Otherwise the methodology used is the same as was used in the text for both calibration methods.

3.1 β

3.1.1 Nelder-Mead

Table A.2 provides the MASE, MAE and RRMSE values for the single variable calibration for the parameter β while holding the value of γ constant at $\frac{1}{8}$. All values for the MASE and RRMSE are below 1 which suggests there is a good fit between the synthetic data and the calibrated model. Additionally the MAE values are small relative to the size of the outbreaks which further suggests a good fit.

Table A.3 provides the distribution of the β values from the calibration for the Nelder-Mead and HMC calibrations. Looking at the median values it can be

Table A.2: Accuracy measures for the Nelder-Mead and HMC Calibrations for β

	Nelder-Mead			HMC		
Scenario	MASE	MAE	RRMSE	MASE	MAE	RRMSE
S1_I0_V0	0.81	35.10	0.75	1.46	32.74	0.78
S2_I50_V0	0.77	29.80	0.81	1.62	28.73	0.90
S3_I100_V0	0.96	16.99	0.31	2.16	23.82	0.58
S4_I0_V50	0.70	10.14	0.49	1.29	13.12	0.75
S5_I50_V50	0.70	9.29	0.49	1.36	10.98	0.77
S6_I100_V50	0.70	8.78	0.25	1.66	11.96	0.39
S7_I0_V80	0.66	2.33	0.72	1.08	2.63	0.84
S8_I50_V80	0.70	1.24	0.67	0.99	1.36	0.71
S9_I100_V80	0.71	0.37	0.45	1.23	1.67	0.57

seen that they range from 0.490 to 0.885 for the Nelder-Mead calibrations. These values are lower than the reference value of 1.5 and lower than the majority of the median values for the β from the dual parameter calibration presented in the main text of the paper. This is likely the model accounting for the changes in behaviours and contact patterns of the agents. When calibrating to two parameters the effective infectious period can compensate for some of the days the agents isolate and do not infect other individuals. However, when the infectious period is held constant at the biological infectious period as it is in this calibration the β value must adjust to account for the changes in the effective infectious period of the agents. This is further shown when looking at the patterns of the β values across vaccination scenarios. As the isolation rate increase while vaccination rates stay the same β decreases: with higher isolation the calibration has to produce lower β to account for fewer infections due to the isolation of infectious agents.

A visualization of the data in Table A.3 can be seen in Figure A.5. The Nelder-Mead calibrations results in large 95% intervals for the scenarios with 0% vaccination and the Figure shows how as isolation increases the calibrated value for β decreases.

3.1.2 HMC

Table A.2 provides the MASE, MAE and RRMSE values for the single variable calibration for the parameter β while holding the value of γ constant at $\frac{1}{8}$. From the table it can be seen that while the RRMSE values are less than 1 for the different scenarios and the MAE values are relatively small, the MRSE values are greater than 1 suggesting that the fit from the calibration might not be the best fit. This follows what was found in the dual parameter calibration.

Looking at the parameter distributions for β in Table A.3, similar to the Nelder-Mead calibration, the HMC results in median β values that are smaller than the reference parameter and smaller than the medians from the dual pa-

Table A.3: Quantiles for the distribution of β for the Nelder-Mead and HMC methods of calibration

Scenario	2.50%	5%	25%	median	75%	95%	97.50%
			Nelder-Mead				
S1_I0_V0	0.29	0.294	0.593	0.6	0.605	0.637	0.638
S2_I50_V0	0.267	0.272	0.516	0.520	0.525	0.551	0.553
S3_I100_V0	0.221	0.226	0.487	0.490	0.492	0.496	0.497
S4_I0_V50	0.755	0.794	0.813	0.818	0.823	0.829	0.830
S5_I50_V50	0.630	0.631	0.638	0.642	0.647	0.654	0.656
S6_I100_V50	0.544	0.545	0.551	0.554	0.558	0.563	0.564
S7_I0_V80	0.868	0.869	0.880	0.885	0.889	0.896	0.897
S8_I50_V80	0.777	0.782	0.796	0.799	0.809	0.836	0.838
S9_I100_V80	0.710	0.711	0.713	0.715	0.718	0.721	0.723
			HMC				
S1_I0_V0	0.644	0.644	0.646	0.647	0.648	0.650	0.650
S2_I50_V0	0.566	0.567	0.568	0.569	0.570	0.572	0.572
S3_I100_V0	0.548	0.549	0.550	0.552	0.553	0.554	0.555
S4_I0_V50	0.864	0.865	0.868	0.870	0.873	0.876	0.878
S5_I50_V50	0.717	0.718	0.721	0.723	0.725	0.729	0.730
S6_I100_V50	0.658	0.659	0.663	0.666	0.668	0.672	0.673
S7_I0_V80	0.928	0.929	0.935	0.937	0.939	0.942	0.944
S8_I50_V80	0.848	0.849	0.852	0.854	0.875	0.885	0.888
S9_I100_V80	0.816	0.817	0.822	0.826	0.829	0.834	0.836

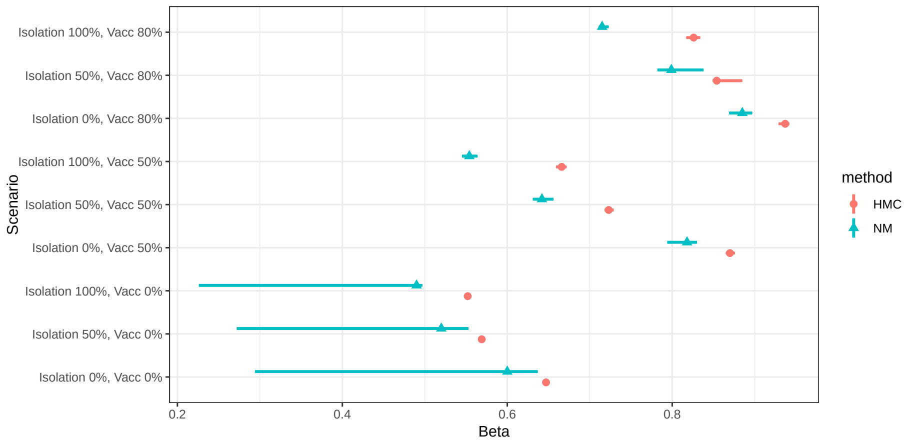

Figure A.5: 95% Quantiles for the Calibrated Model Parameter β.

Table A.4: Accuracy measures for the Nelder-Mead and HMC Calibration of the infectious period

	Nelder-Mead			HMC		
Scenario	MASE	MAE	RRMSE	MASE	MAE	RRMSE
S1_I0_V0	0.96	68.95	1.15	1.57	35.19	0.82
S2_I50_V0	1.02	54.41	1.20	1.71	30.33	0.94
S3_I100_V0	1.63	65.76	0.77	1.92	21.16	0.48
S4_I0_V50	0.79	24.84	1.31	1.27	12.82	0.80
S5_I50_V50	0.83	19.31	1.16	1.54	12.40	0.84
S6_I100_V50	0.83	16.27	0.49	1.53	10.92	0.39
S7_I0_V80	0.83	4.16	1.36	1.07	2.60	0.83
S8_I50_V80	0.72	1.85	1.08	0.99	1.36	0.71
S9_I100_V80	0.71	1.50	0.68	1.33	1.73	0.57

rameter calibration. The medians range from 0.552 to 0.937 and the lower values tend to be in the scenarios with higher isolation levels. The HMC calibration also has a smaller distribution of parameters for each scenario compared to the Nelder-Mead. This can be seen in Figure A.5.

3.2 γ

3.2.1 Nelder-Mead

Table A.4 gives the MASE, MAE and RRMSE for the calibrations for γ while holding β constant. Although there are some scenarios that have MASE and/or RRMSE values above 1, the majority of scenarios have a values less than 1 suggesting a good fit. The MAE values while small, are larger compared to the calibration for β in Table A.2.

The distribution of the infectious period ($1/\gamma$) from the calibrations can be found in Table A.5. The Nelder-Mead calibration results in relatively low infectious periods for all scenarios, medians between 1 and 2.09 days. The biological infectious period of measles is approximately 8 days. Although some of the scenarios in the dual parameter calibration presented in the main paper had small infectious periods from the Nelder-Mead calibration (scenarios with 50% vaccination or 100% isolation). There were a number of scenarios that had more realistic values for the infectious period compared to the biological infectious period. This suggests that the dual parameter calibration might be better at capture the infectious period values due to its ability to account for the correlations between β and γ.

Figure A.6 shows the 95% quantiles for the infectious period. Looking at the Nelder-Mead results we can see that for the scenarios with 50% and 80% vaccination as isolation increases, the infectious period also increases, however this is only by a small amount and the intervals overlap in particular for the 50% vaccination scenario. For the scenario with 100% vaccination, the infectious period decreases as isolation increases.

Table A.5: Quantiles for the distribution of infectious period for the Nelder-Mead and HMC methods of calibration

Scenario	2.50%	5%	25%	median	75%	95%	97.50%
			Nelder-Mead				
S1_I0_V0	1.94	1.95	2.01	2.06	2.10	2.18	2.20
S2_I50_V0	1.57	1.58	1.62	1.64	1.66	1.70	1.72
S3_I100_V0	1.00	1.00	1.00	1.00	1.00	1.00	1.00
S4_I0_V50	1.83	1.87	1.94	1.97	2.01	2.08	2.10
S5_I50_V50	1.97	1.99	2.03	2.06	2.09	2.13	2.14
S6_I100_V50	2.01	2.03	2.06	2.09	2.152	2.17	2.18
S7_I0_V80	1.26	1.27	1.29	1.30	1.32	1.34	1.35
S8_I50_V80	1.36	1.37	1.38	1.39	1.41	1.42	1.43
S9_I100_V80	1.42	1.42	1.43	1.44	1.45	1.46	1.47
			HMC				
S1_I0_V0	2.06	2.06	2.07	2.07	2.08	2.08	2.09
S2_I50_V0	1.84	1.84	1.84	1.84	1.85	1.85	1.85
S3_I100_V0	1.77	1.77	1.77	1.78	1.78	1.79	1.79
S4_I0_V50	3.36	3.36	3.38	3.45	3.46	3.56	3.57
S5_I50_V50	2.73	2.73	2.75	2.82	3.15	3.27	3.28
S6_I100_V50	2.60	2.61	2.62	2.63	2.78	2.80	2.80
S7_I0_V80	1.06	1.06	1.07	1.07	1.07	1.08	1.08
S8_I50_V80	1.12	1.13	1.14	1.17	1.17	1.18	1.18
S9_I100_V80	1.20	1.20	1.21	1.22	1.22	1.23	1.23

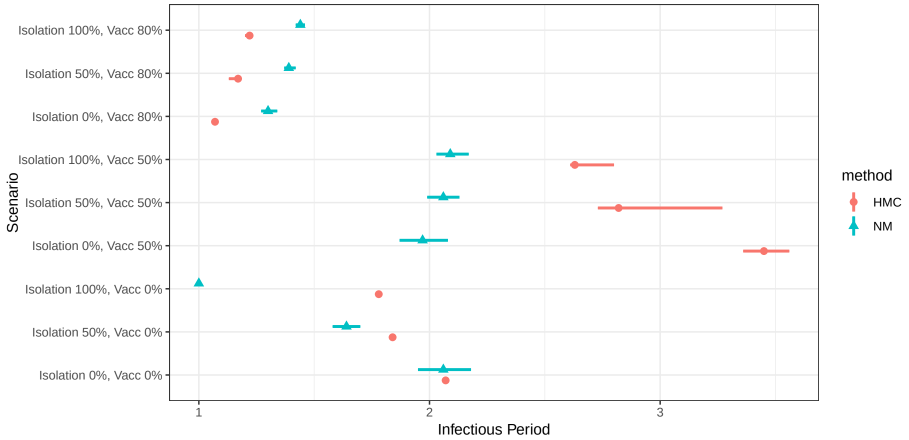

Figure A.6: 95% Quantiles for the Calibrated Model infectious period.

3.2.2 HMC

The MASE, MAE and RRMSE for the HMC calibrations for γ can be found in Table A.4. While the RRMSE and MAE values are low for the majority of scenarios, the MASE values are above 1.0. This suggests that the calibrated fit might not be the best. However, the MAE values are lower for the HMC compared to the Nelder-Mead calibrations.

Table A.5 and Figure A.6 show the distribution of the calibrated infectious period parameter across all scenarios. The median infectious periods for the HMC calibration while still low ranging from 3.45 to 1.07, for the scenarios with 0% and 50% vaccination the median infectious periods are higher than the Nelder-Mead values. For the 0% and 50% vaccination scenarios, the infectious period decreases as isolation increases while in the 80% vaccination scenarios there is a slight increase in the infectious period as isolation increases.
